# White Matter Changes: New Perspectives on Imaging, Clinical Aspects, and Intervention

**DOI:** 10.4061/2011/841913

**Published:** 2012-02-26

**Authors:** Sofia Madureira, Ana Verdelho, Leonardo Pantoni, Philip Scheltens

**Affiliations:** ^1^Department of Neurosciences and Mental Health, Hospital de Santa Maria, University of Lisbon, 1649-004 Lisbon, Portugal; ^2^Department of Neurological and Psychiatric Sciences, University of Florence, 50121 Florence, Italy; ^3^Department of Neurology and Alzheimer's Center, VU University Medical Center, 1007 MB Amsterdam, The Netherlands

In the last three decades, the development of more sensitive techniques used in the assessment of *in vivo *brain functioning, such as computed tomography (CT) scan and magnetic resonance imaging (MRI), has contributed to identify cerebral changes that are either associated with clinical features (dementia and cognitive impairment) or described in normal subjects [1].

The presence of age-related white matter changes (ARWMCs) is described with the increasing age and the presence of vascular risk factors as the major determinants for these findings [2–4], but also in subjects over 50 years old [5].

The research of the clinical significance of ARWMC has clearly demonstrated the association between the presence of white matter changes and cognitive impairment, behavioural changes, urinary disturbances, and gait difficulties. However, and despite the increasing interest in this field, the interpretation of the data and explanation of these mechanisms are still under discussion.

The aim of the current special issue was to explore the heterogeneity of the areas of impact of the ARWMC, by including different and less frequent approaches that allow for a more comprehensive picture.

Focused in this general goal, we included a variety of papers ranging from an overview of the pathophysiology, assessment, and clinical manifestations of ARWMC, as well as a complete revision of proposed treatments—illustrated by the review of Y. Y. Xiong and V. Mok—to the proposal of a mouse model of chronic cerebral hypoperfusion as a possible tool on the explanation of the molecular pathology of white matter lesions—presented by M. Ihara and H. Tomimoto. With the same perspective, we included the paper of C. Sierra et al. that presents a nice review about the relation between hypertension and long-standing effect in the cerebral white matter. L. Yang reviewed issues related to brain plasticity and its possible relation to age-related white matter changes, a topic of novel interest. Also of interest is the work of A. Xekardaki et al. that explores the use of diffusion tensor imaging in bipolar disorder.

Besides the review papers, 8 original articles were included in this special issue and are grouped in three blocks. The first one tried to focus on the anatomical correlations and clinical features that are related with the presence and progression of white matter changes—cognitive changes (memory), motor dysfunction, late-onset depression, and health-related quality of life. E. T. Schulze et al. provided us with an innovative approach by using multifaceted imaging techniques on the evaluation of brain functional changes during working memory tasks. In a less addressed field of research, M. Viana-Baptista et al. reflected on the importance of frontal white matter lesions for the compromise of motor performance. J. A. Brommelhoff et al. also pointed out that late-onset depression could be a process different from neurodegenerative changes and describe no differences in frontal lobe deep or subcortical white matter between Alzheimer's patients with or without late-onset depression. A. M. Grool et al. discussed the relation between progression of WMC volume and health-related quality as part of the large SMART Study adding data to the existing body of literature on the role of progression of white matter lesions on decline in mental functioning in patients with symptomatic vascular disease. A very interesting paper by V. Rajagopalan et al. reports innovative imaging data in amyotrophic lateral sclerosis (ALS) patients with predominant upper motor neuron (UMN) signs.

A second section, mainly addressed to MRI analysis and measurement of white matter changes, is represented in the paper by S. D. Smart et al., who present the validation of automated WMH segmentation. These authors showed that automated segmentation of the brain provides good to very good estimates of the volume of the affected white matter, which will enable further research into the role of ARWMC in various diseases.

Finally, the last block of papers refers to different basic sciences, reflected both on the interesting paper of H. Chen et al. that evidences reductions in fiber tract volumes and loss of axonal neurofilaments, and on the paper by B. D. James et al., that brought surprising findings on genetic precursors of dementia and led us into an open discussion for possible future directions.

*Sofia Madureira* *Sofia Madureira* *Ana Verdelho* *Ana Verdelho* *Leonardo Pantoni* *Leonardo Pantoni* *Philip Scheltens* *Philip Scheltens*
